# Impact of Individual, Household and Community Characteristics on Children's Nutritional Indicators

**Published:** 2014-06

**Authors:** Lucky Mokgatlhe, Maria S. Nnyepi

**Affiliations:** ^1^Department of Statistics, University of Botswana, P/Bag 0022, Gaborone, Botswana; ^2^Department of Family and Consumers Sciences, University of Botswana, P/Bag 0022, Gaborone, Botswana

**Keywords:** Community, Fixed effect, Intra-class correlation, Multilevel analysis, Multistage cluster sampling, Random effect, Botswana

## Abstract

This study analyzed WHO-standardized nutritional indicators of children from selected households within communities that were sampled from all districts of Botswana. Data from the 2007 Botswana Family Health Survey were fitted into multilevel models that seek to account for variability due to the macro- and micro-units that have been hierarchically selected. This allowed for estimation of different levels of intra-class correlations while simultaneously assessing the model-fit by accounting for the influence on the nutritional indicators due to the fixed variables attributable to these macro- and micro-units. The results show that variation in nutritional status of under-five children in Botswana is a function of characteristics of the households and communities within which they live. As much as 17% of variation is due to differences in the communities and households. Economic status of households holds an important key in predicting the nutritional status of children.

## INTRODUCTION

Owing to the high prevalence of malnutrition worldwide, children aged 0-5 year(s) have received much programming attention. The focus of such interventions is on reducing the prevalence of malnutrition; thus, these interventions give children a fair chance of survival, growth, and development. Typically, most interventions target all under-five children. Occasionally, child-level factors (birth, weight, age) and maternal factors (maternal nutritional status during pregnancy) are used in further refining the targeting of children at higher risk of poor growth. Also, more efforts are put in targeting children during developmental windows where interventions have the most impact, such as age for complementary feeding and reaching children who are not reached by high-impact interventions. Commendable as these interventions are, there is a growing evidence that commands programmers’ attention to the influence of the household and community environments on child nutrition (2-5). In 2007, prevalence of malnutrition in Botswana was reported to be 11.2% while stunting was 26%. The Government has, since independence, championed the fight against malnutrition. There is a provision to give supplementary feeding to under-five children whose nutritional status is classified as malnourished. Despite all endeavours to achieve the desired goals, the results have been excruciatingly slow. This is partly because the approach is not proactive as it fails to pre-assess children who do not receive supplements, although presently, they may be well-nourished. The households or communities they reside in pre-dispose them to malnourishment. It is desirous that an appropriate mechanism be devised that can enable identification of environments that expose under-five children to these nutritional ills. For these reasons, there is need to fully consider the determinants of childhood malnutrition more comprehensively and in the context of both household and community environments. This requires the use of study approaches that provide for examination of the influence of different living environments on child growth factors. The objective of this study is to fit Botswana data into multilevel models to assess the impact of individual, household and community factors on children's nutritional status. The results should offer evidence-based intervention alternatives. In our literature search, we could not come across any study that has endeavoured to fit Botswana data into multilevel models.

## MATERIALS AND METHODS

In social sciences where data are collected from randomly-selected samples as a way of reducing bias, multistage cluster sampling of grouped sampling units selected in a hierarchical manner is used. This is a preferred method because of its cost-effectiveness. In analyzing data from this type of study design, one needs to account for possible correlation in the characteristics of observations drawn from a common cluster, hence, calling multilevel analysis. The method calls for a distinction to be noted between aggregated levels and individual levels (6). The method is subsequently referred to as within-group and between-group regression (7-8) in the discussion of regression intercepts and slopes of one level as an outcome on the higher level. Multilevel analysis, as a method of analyzing data with pattern-complex variability with a focus on nested sources of variation is described by Snijders and Bosker (9). The method utilizes mixed models (random and fixed effect) and, hence, ably deals with dependency (correlated responses) that emanates from using multistage cluster sampling. One of the assumptions in linear modelling is that of independence between observations but, in this case, the assumption is violated since households in selected communities have their probability of selection being enhanced once such community has been selected. Moreover, responses within households or even communities are likely to be correlated, creating some dependency. A mixed model, henceforth, is able to deal with fixed effects at both community and individual levels while quantifying the variance component contributed by a sample of groupings (random effect) of responses at the community level. The arrival of general mixed linear model (10) as a statistical model, therefore, crystallized the analysis of variance, even though the mixed models seem to have been used prior to this (11). Mixed linear models with different link functions that include probit, logit, and inverse log known as generalized mixed linear models were subsequently used by Kachman (12).

### Health survey data

The authors used a nationally-representative cross-sectional data from 2007 Botswana Family Health Survey (BFHS) to determine the individual-, household- and community-level effects on the nutritional status of children aged 0-5 year(s). Reference is made of the Central Statistics Office (CSO) 2009 study report for the details of the multistage sampling procedures used (13). The survey is conducted once in every 10 years. The data were collected using a two-stage process, by first sampling enumeration areas (EAs) proportionately from each district based on their population-sizes as established during the 2001 census survey. An EA is defined as an area consisting of an average of 100-250 households that share some common amenities, like street, shops, clinic, etc., thus constituting a community. The second stage entailed a systematic selection of households within each randomly-selected EA, guided by sampling frames available for each EA. Households were further stratified by whether these were drawn from EA that belonged to either rural (small villages, lands, and cattle posts/farms), ‘urban villages’, or cities/towns, for their regional location. ‘Urban villages’ are operationalized as settlements with a population of 5,000 inhabitants and naturally consist of several EAs or communities.

Once a household was randomly selected, information and nutritional and demographic characteristics of all eligible children in the household was collected as reported by a parent or legal guardian. Household data were collected using the household questionnaire. The questionnaire was developed in collaboration with key stakeholders, inclusive of the government ministries and development partners. These were pilot-tested prior to the use. The questionnaire had several sections, inclusive of sociodemographic characteristics, housing characteristics, employment status, and other economic characteristics, education, and social characteristics. The survey instruments are available in the BFHS report (13). Data pertaining to anthropometric measurements were also taken. Weight and height measurements were collected by trained research assistants. Children's weights were measured to the nearest 0.1 kg, using Seca Scales, Model 871. Standing height was measured for all children over two years of age while length was measured for all those who were aged 2 years or younger. The length/height was measured to the nearest 0.1 cm.

For this study, the child-level variables selected for analysis were age, gender, birthweight, and whether the child has ever been breastfed. The household-level variables of interest were caregivers’ characteristics (gender, age, education, and employment status) and household wealth status. The household wealth scores were created using factor analysis for household assets and access to basic services, inclusive of the ownership of house, quality of housing, access to water, toilet facilities, sources of cooking and lighting energy, and ownership of assets. The outcome indicators of children's nutrition were z-scores of weight-for-age (WAZ), weight-for-height (WHZ), and height-for-age (HAZ), which were standardized using the WHO Anthro software.

Since children within a household can only belong to that household and a given household is found in only one community, these units are nested within each other. The sample consisted of 393 geographic enumeration areas (EAs), 7,860 households, and 2,822 children aged 0-5 year(s).

### Statistical analysis

Total variation in the children's characteristics can be attributed to two sources: variation among children and variation due to the cascading sampling levels. The use of multilevel approach in explaining malnutrition among the under-five children was applied to the Nigerian Demographic and Health Survey data on under-five children by Uthman (14), using a generalized linear model with a logit link function. However, dichotomizing of the nutrition status into whether one is malnourished or not is only helpful when the focus is on understanding undernutrition; it does not simultaneously address the issue of obesity which is fast becoming a topical issue in Botswana (15).

Because of the nature of design used in collecting the survey data, the sample is potentially clustered on three levels: individual (Level 1), household (Level 2), and community (Level 3). In studying the individual, household and community effects on the nutritional status of children aged 0-5 year(s), the authors analyzed the data by developing three simple variance components regression models on the standardized scores of the response variables. In the first model, we assessed whether each nutritional indicator varies across individuals, households, and communities by fitting the three-level random intercept model with no observed covariates (the empty model). The percentage of total variation in the dependent variables attributable to clustering at levels was referred to as the community intra-class correlation coefficient and was used as a measure of contextual effects. The Level-3 intra-class correlation expressing the likeness of children from the same community were approximated by 
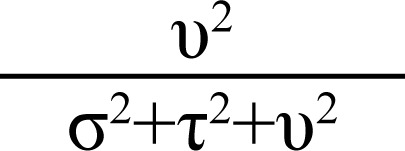
 where *v*^2^ is the community-level variance, τ^2^ is the household-level, and σ^2^ is the individual-level variance. In the second model, household-level factors were added to the empty model while the third model included all household factors together with individual factors and a general mixed linear model (16,17) of the form fitted:


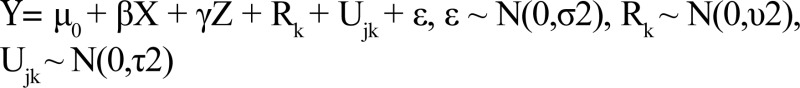


where Y is a vector of responses from individuals (Level 1); μ_0_ is the population grand mean; β is a vector of parameters associated with individual characteristics X, given in a matrix form γ is a vector of parameters associated with household characteristics Z, also given in a matrix form R_k_ is the Level-3 effect of the k^th^ community while U_jk_ is the Level-2 effect of the j^th^ household nested within k^th^ community; and ϵ is the error term which is assumed to be normally distributed. Each child's response within a household deviates from the true mean by some value ϵ while households differ from one another as reflected by U_jk_ and R_k_, which, in this case, are all treated as random effects. To establish whether households’ socioeconomic status was an important predictor of height-for-age, weight-for-age, and weight-for-height, the wealth scores were taken as fixed effects. Three cascading mixed models were fitted on each of the three response variables, and all these were done using SPSS (version 20.0) (18) and R (version 2.14.1) software.

## RESULTS

### Sociodemographic variables

The survey covered a total of 2,822 children aged less than five years but complete information on the basic demographic characteristics of children was missing in some variables. Of the 2,822 children sampled, only 2,719 had information on their ages recorded. With respect to other variables, fewer than 2,719 children had complete information on each of these variables.

**Figure 1.**
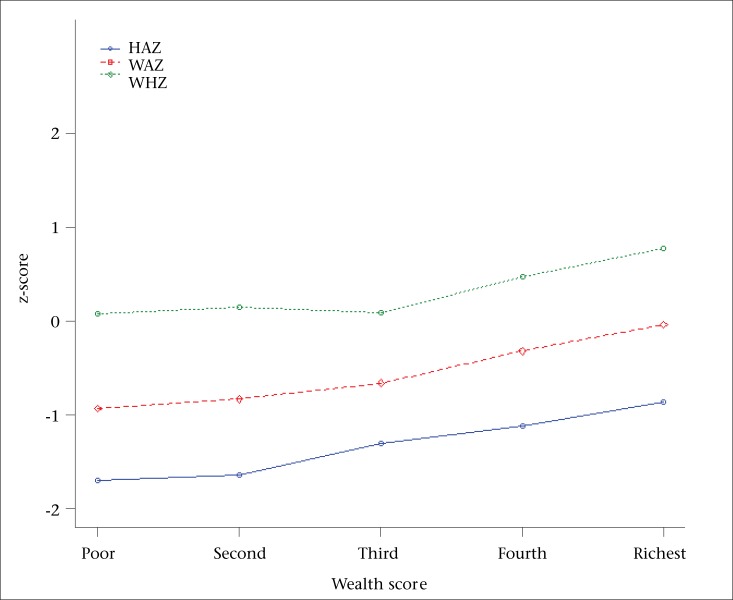
A profile plot of means on nutritional indicators

Figure 1 shows that, in general, the average nutritional indicators tended to improve with higher levels of wealth in the household. Using a Student's *t*-test on the means for age, height, BMI, and weight, no statistical difference was observed between male and female children on the first three variables, except for weight where males markedly displayed higher average weight (p<0.001) (Table 1). Similarly, using a homogeneity test for equality of proportions, no difference between genders of children sampled from various households with different wealth scores was found.

**Table 1.** Sociodemographic characteristics of the participating under-five children and their households

Variable	Male		Female		*t*-test (95% CI)
	n	Mean (SEM)	n	Mean (SEM)	
Age (months)	1,368	28.58 (0.454)	1,351	28.75 (0.456)	0.268 (-1.09,1.43)
Height (cm)	1,302	84.13 (0.413)	1,291	83.15 (0.430)	−1.642 (-2.15,0.19)
Weight (kg)	1,324	11.82 (0.099)	1,315	11.33 (0.097)	−3.558 (-0.76,-0.22)
BMI (kg/m^2^)	1,298	16.88 (0.106)	1,288	16.68 (0.128)	−1.232 (-053,012)
Wealth score	n	Prop (SEP)	n	Prop (SEP)	
Poorest	335	0.250 (0.024)	344	0.254 (0.023)	
Second	315	0.235 (0.024)	300	0.221 (0.024)	
Third	256	0.191 (0.025)	269	0.198 (0.024)	
Fourth	253	0.189 (0.025)	238	0.175 (0.025)	
Richest	183	0.136 (0.025)	206	0.152 (0.025)	

χ^2^=2.542, df=4, (p=0.637); CI=Confidence interval; Prop=Estimated proportion; SEM=Standard error of mean; SEP=Standard error of proportion

### Modelling height-for-age

A general linear model shown below was used in assessing the effect of household and community effects on children's height-for-age. The model, referred to as Model I, only had a three-level random intercept.

**Figure 2.**
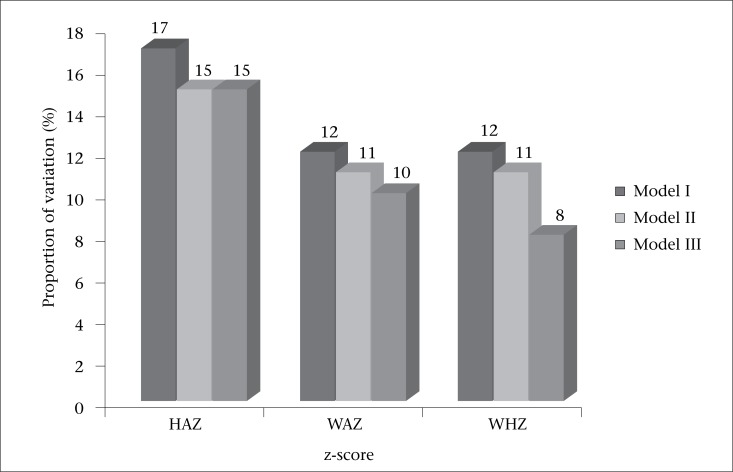
Proportion of variation (%) attributed to households nested within a community for Model I-III

The intra-class correlation at Level 3, expressing the likeness of height-for-age z-scores for children in the same community, is 2% 
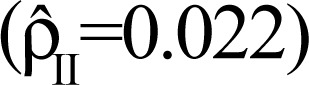
 while that for children in different households within the same community is 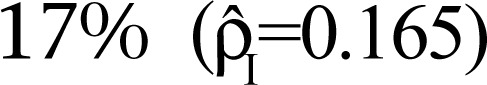 and is significantly different from zero. This shows that, while ignoring other factors, 17% of variation in height-for-age is attributed to differences in households within a community, (Figure 2). Considering the Level-2 model, we see that the intra-class correlation, which expresses likeness in height-for-age for children in different households within a community, is estimated at 0.99. This suggests that households within a community contribute more variability in the height-for-age z-scores than when community groupings are considered alone (Table 2).

The second model (Model II) was built around Model I consisting of the same parameters but now including factors measured at the household level while controlling for wealth indicator, which is considered a fixed effect.

Whereas the individual unit variance in this model is estimated to be 
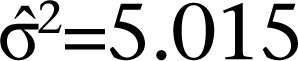
 and is significantly different from zero, the estimated between-community effect variance component is only 
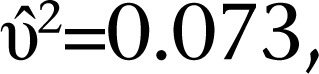
 with an estimated intra-class correlation of 
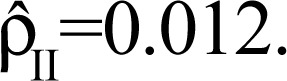


When controlling for wealth score of a household, education of caretaker and age of caretaker, the estimated intra-class correlation between children in different households within a community is 15% (Figure 2). The effect of different levels of economic status for different households significantly affected height-for-age of children in varying ways, with children from well-off households showing comparatively better height-for-age z-scores. Comparing children in households where a caregiver had secondary education, other educational levels lower than secondary did not affect the height-for-age z-score of the child. The explained Level-1 proportion of variance (analogous to R^2^) (9) by introducing fixed effects at the household level to an empty model was 1%. Despite this low proportion of explained variance, fixed effect variables were essential in this model as evidenced by the change of 2,526.65 in the model deviance. Model III consisted of all the fixed effects at the individual and the household levels plus the random effects used in earlier models as shown below, yielding increased intra-class correlation for both between-households nested within communities and between-communities at 15% and 5% respectively.

**Table 2.** Parameter estimation using three-level model on height-for-age

Fixed effects	Model I	Model II	Model III
	Estimate (95% CI)	Estimate (95% CI)	Estimate (95% CI)
Intercept	−1.370 (-1.47,-1.26)	−0.740 (-1.21,-0.27)	−2.200 (-3.19,-1.21)
Individual level			
Age (completed months)			
0-11	−	−	0.629 (0.09,1.17)
12-23	−	−	−0.351 (-0.73,0.02)
24-35	−	−	−0.528 (-0.87,-0.19)
36-49	−	−	−0.340 (-0.70,0.02)
48-59	−	−	−
Gender			
Male	−	−	−0.313 (0.07,0.56)
Female	−	−	−
Birthweight (kg)	−	−	0.471 (0.22,0.72)
Household level wealth	
Poorest	−	−0.793 (-1.20,-0.39)	−0.816 (-1.27,-0.35)
Second	−	−0.721 (-1.10,-0.34)	−0.560 (-0.99,-0.13)
Third	−	−0.472 (-0.86,-0.09)	−0.603 (-1.03,-0.18)
Fourth	−	−0.345 (-0.73,0.04)	−0.412 (-0.83,0.00)
Richest	−	−	−
Age of caretaker (completed years)			
<25	−	−0.083 (-0.53,0.36)	−0.190 (-0.69,0.31)
25-49	−	0.027 (-0.36,0.41)	0.024 (-0.40,0.45)
>49	−	−	−
Education of caretaker			
No education	−	−0.061 (-0.48,0.36)	0.082 (-0.39,0.55)
Primary	−	−0.216 (-0.50,0.06)	−0.188 (-0.51,0.14)
Secondary	−	−	−
Random effect	Model I	Model II	Model III
Community (Var.)	0.130 (0.04,0.42)	0.073 (0.00,0.76)	0.240 (0.09,0.66)
Household (Community) (Var.)	0.985 (0.64,1.52)	0.860 (0.47,1.56)	0.673 (0.24,1.86)
Residual	4.837 (4.41,5.29)	5.015 (4.51,5.57)	3.508 (2.90,4.25)
VPCH (%)	16.5	14.5	15.2
Deviance	11,950.02	9,423.37	5,009.69

Var.=Estimated variance; VPCH=Variance partition coefficient-household

The child-to-child variation is estimated to be 
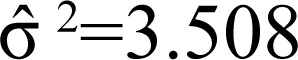
 while the explained proportion of variance as result of including all fixed effects into an empty model was R^2^=26%. The model deviance value has further reduced by 6,940 from the original empty one.

It is clear that, at the child level, height-for-age scores were influenced by age of the child. Compared to children aged 4 years or more, those less than one year had better height-for-age z-scores while the rest of the groups were comparatively worse-off. Children with elevated birthweight were significantly more likely to have higher height-for-age z-scores than those weighing less at birth while males were significantly disadvantaged than females. While controlling for individual traits of children, the major source of variation in height-for-age attributable to household is wealth status of household.

### Modelling weight-for-age

The empty model I (Table 3), which only has a random intercept and three-level model, was fitted in a hierarchical order for the weight-for-age variable. The estimated error term variance of 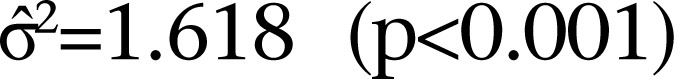 was obtained, which measured within-subjects variation while the Level-3 (community) components yielded a variance of 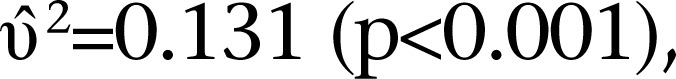 and the Level-2 model (households within community) yielded a variance of 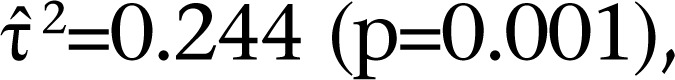 all of which were significantly different from zero. The likeness of weight-for-age z-scores (Figure 2) for children between households in the same community was 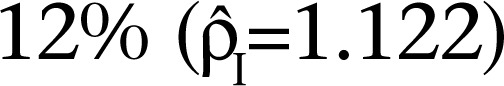 while the likeness between children in different communities was only 7% 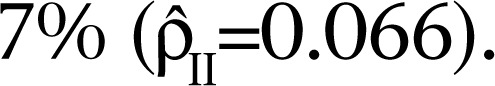 Once again, this demonstrates the variation that exists between households in different communities, making it crucial to analyze children's nutritional growth at the household level within communities.

**Table 3.** Parameter estimation using three-level model on weight-for-age

Fixed effects	Model I	Model II	Model III
	Estimate (95% CI)	Estimate (95% CI)	Estimate (95% CI)
Intercept	−0.591 (-0.66,-0.52)	0.103 (-0.13,0.33)	−2.584 (-3.05,-2.12)
Individual level			
Age (completed months)			
0-11	−	−	0.603 (0.41,0.80)
12-23	−	−	0.350 (0.16,0.54)
24-35	−	−	0.112 (-0.08,0.30)
36-47	−	−	0.075 (-0.12,0.27)
48-59	−	−	−
Birthweight (kg)	−	−	0.800 (0.68,0.92)
Gender			
Male	−	−	−0.167 (-0.28,-0.05)
Female	−	−	−
Household level wealth	
Poorest	−	−0.780(-0.98,-0.58)	−0.651 (-0.88,-0.43)
Second	−	−0.725 (-0.92,-0.53)	−0.603 (-0.81,-0.40)
Third	−	−0.600 (-0.80,-0.41)	−0.525 (-0.73,-0.32)
Fourth	−	−0.293 (-0.49,-0.10)	−0.234 (-0.44,-0.03)
Richest	−	−	−
Age of caretaker (completed years)		
<25	−	−0.208 (-0.42,0.01)	−0.272 (-0.51,-0.03)
25-49	−	−0.098 (-0.28,0.09)	−0.165 (-0.37,0.04)
>49	−	−	−
Education of caretaker			
No education	−	−0.292 (-0.49,-0.09)	−0.142 (-0.37,0.09)
Primary	−	−0.119 (-0.26,0.02)	−0.046 (-0.20,0.11)
Secondary	−	−	−
Random effect	Model I	Model II	Model III
Community (Var.)	0.131 (0.08,0.21)	0.075 (0.04,0.15)	0.098 (0.05,0.18)
Household (Community) (Var.)	0.244 (0.14,0.43)	0.206 (0.11,0.40)	0.170 (0.07,0.43)
Residual	1.618 (1.48,1.77)	1.611 (1.47,1.76)	1.501 (1.34,1.68)
VPCH (%)	12.2	10.9	9.6
Deviance	9,252.11	9,092.35	7,091.51

Var.=Estimated variance; VPCH=Variance partition coefficient-household

In Model II, factors measured at the household level, i.e. traits pertaining to caregiver as well as household wealth score as fixed effects, are included. The within-group variance reduced slightly while the community effect variance was reduced to 0.075 (p=0.003); hence, an estimated intra-class correlation of 
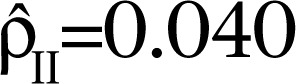
 implied that the likeness in terms of weight for children in different communities was only 4% while that between children in different households within a community was 11% (Table 2). As for the fixed effect, while controlling for random effects, we observed that, at the household level, wealth status of household and education level of caregivers influenced changes in weight-for-age z-scores. Children in households that reported wealth status lower than those identified as the richest households and a primary or lower education level compared to secondary level for caregivers tended to have lower weight-for-age z-scores. Even though these variables were statistically related to weight scores, their contribution alone to the model was minimal. The explained Level-1 proportion of variation due to variables at the household level was 5%. The model-fit deviance change was 159.76.

Considering the child-level characteristics (Model III), children who had a higher birthweight and were female were, on average, more likely to have higher weight-for-age z-scores. In benchmarking on children who were four years of age or older, younger children had better weight-for-age z-scores, even though only those aged less than 24 months were statistically different (each with p<0.001) (Table 3). Of interest to note is that, inclusion of child's characteristics in the model changed the effect that caretaker's age has; a child whose caretaker was aged less than 25 years tended to have lower weight-for-age z-score. Whereas the total estimated variance for this model was reduced, the likeness of weight-for-age z-scores for children in different communities increased slightly to 6% while that of children in different households within communities remained at 10%. Thus, the explained Level-1 proportion of variation due to inclusion of both household level and child's characteristics in the model was 11%. We also noted a drastic change of 2,000.84 in the model deviance value as a result of including child's characteristics in the model.

### Modelling weight-for-height

An approach similar to the ones used for the two previous variables was used in analyzing weight-for-height which is a good indicator for wasting among children. The empty Model I (Table 4) yielded a total variance of 4.439, revealing a moderate household-to-household variation in relation to weight-for-height z-scores. A variance of 0.511 (p=0.002) was attributed to between-households nested within a community variation with an intra-class correlation value of 
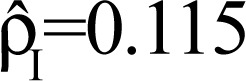
 while that between communities was only 
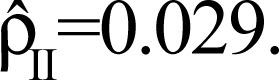


Model II was obtained by adding wealth status variable as fixed effect and other caregivers’ attributes measured at the household level (age of caregiver and education of caregiver). It is clear that, from the included fixed effects, weight-for-height z-scores were significantly influenced by wealth status score of the household only, though minimally. Once again, compared to rich families, all other children from families with economic status below the rich were disadvantaged and, hence, were more susceptible to wasting than the rich. The estimated total variance has reduced slightly to 4.402 while the between-community variation reduced down to 
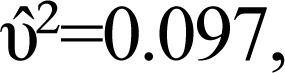
 giving a between-community intra-class correlation of 
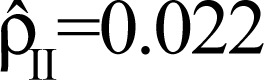
 and that between households in a community reduced to 
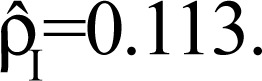
 A change of 86.92 in model deviance was recorded as a result of the inclusion of fixed effect variables. The explained Level-1 proportion of variation due to fixed-effect variables is less than 1%.

Finally, Model III encompassed variables that measured the characteristics at the individual level of age, birthweight, and gender of the child. While controlling for other variables, comparing each category with the richest family status, the average difference between the richest and the fourth category of wealth status was not statistically significant while that between the richest and the third category (median economic status) showed the largest difference. Furthermore, comparing older children (48-59 months) with all other younger age-groups showed that a younger child had better weight-for-height z-score. Meanwhile, on average, those children born with higher birthweight tended to have better weight-for-height z-score. Total estimated variance has decreased slightly while individual estimated variance was accentuated to 
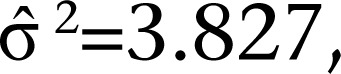
 lowering the intra-class correlation between households in the same community to 8%. Despite a significant reduction in the model deviance (2,334.80), the model-fit was moderate as the fixed effect variables explained 6% proportion of variation in the weight-for-height z-scores.

**Table 4.** Parameter estimation using three-level model on weight-for-height

Fixed effects	Model I	Model II	Model III
	Estimate (95% CI)	Estimate (95% CI)	Estimate (95% CI)
Intercept	0.271 (0.18,0.36)	0.712 (0.35,1.07)	−1.616 (-2.42,-0.81)
Individual level			
Age (completed months)			
0-11	−	−	0.709 (0.37,1.04)
12-23	−	−	0.867 (0.53,1.20)
24-35	−	−	0.416 (0.09,0.74)
36-47	−	−	0.437 (0.10,0.77)
48-59	−	−	−
Birthweight (kg)	−	−	0.561 (0.36,0.76)
Gender			
Male	−	−	−0.023 (-0.22,0.18)
Female	−	−	−
Household level wealth			
Poorest	−	−0.696 (-1.00,-0.38)	−0.540 (-0.91,-0.17)
Second	−	−0.644 (-0.94,-0.35)	−0.432 (-0.77,-0.09)
Third	−	−0.593 (-0.99,-0.39)	−0.573 (-0.92,-0.23)
Fourth	−	−0.347 (-0.65,-0.05)	−0.075 (-0.42,0.27)
Richest	−	−	−
Age of caretaker (completed years)		
<25	−	0.031 (-0.31,0.37)	−0.127 (-0.56,0.31)
25-49	−	0.088 (-0.20,0.37)	−0.061 (-0.44,0.32)
>49	−	−	−
Education of caretaker			
No education	−	−0.081 (-0.39,0.23)	0.036 (-0.36,0.43)
Primary	−	0.059 (-0.16,0.28)	0.120 (-0.14,0.38)
Secondary		−	−
Random effect	Model I	Model II	Model III
Community (Var.)	0.130 (0.05,0.36)	0.097 (0.03,0.34)	−
Household (Community) (Var.)	0.511 (0.28,0.95)	0.499 (0.26,0.94)	0.355 (0.12,1.08)
Residual	3.798 (3.47,4.16)	3.806 (3.48,4.17)	3.827 (3.42,4.28)
VPCH (%)	11.5	11.3	8.4
Deviance	11,010.65	10,923.73	7,068.86

Var.=Estimated variance; VPCH=Variance partition coefficient-household

## DISCUSSION

Whereas certain variables at the household and individual level influenced differences in child's nutritional indicators, part of this variation in this study is attributable to the differences in households and communities. Consistent and in agreement with observations from other authors, children in households with low resources fared worse compared to those in well-resourced households (19). Unlike household wealth, other household-level variables, like different levels of caregiver's age and education, influenced some child growth indicators more than these did in others. Similarly, at the individual child level, the age of the child influenced all the variables while the influence of birthweight and gender on the child's growth depended on the nutritional indicator being considered.

These findings confirm observations by others (20) that the child-level factors do explain some of the variation in the risk of child nutrition and should, therefore, be considered in targeting the needy. However, there are household- and community-level factors that also should be considered on their own merit (3,5,19,21,22). Significant among these are variations in households, especially in terms of household wealth status and variations in environments at the community level. While it is, indeed, easier to target specific children based on their age, birthweight, and current growth indicators, this is unlikely to address household-level environments that led to the poor nutritional status of the affected children. Targeting affected children is necessary from a recuperative perspective but the benefits are short-lived and are unlikely to correct the non-supportive household and community environments or minimize their adverse effect on other children in the future.

Evidence from this study and others (19) support the use of household asset profile to identify and target households with children at risk of poor growth indicators for interventions. Targeting households is consistent with the communal manner in which households in some societies typically disburse their resources. Therefore, rather than targeting the child, the interventions would target the household as a unit and deliver interventions that empower households to care for the children. Such interventions are less prone to leakages (23). Further, unlike the targeting of children, which is normally narrowed down to the child and the caregiver, approaches that target households as a unit are likely to engage the community because these influence environments where households within a community share.

## ACKNOWLEDGEMENTS

The researchers wish to thank Statistics Botswana as the custodian of the secondary data used in this research for analysis. They further express gratitude to the UNICEF (Botswana Office) for the logistical support which culminated in a technical report that formed the basis for this manuscript and the two colleagues Dr. S. Maruapula and Dr. K. Gobotswang from the Department of Family and Consumers Sciences.
